# Long-Term Dietary Supplementation with Quebracho-Derived Condensed Tannin in Growing–Finishing Pigs: Effects on Growth Performance, *Ascaris suum* Infection, Intestinal Bacterial Community Structure and Meat Quality

**DOI:** 10.3390/ani16213487

**Published:** 2026-11-04

**Authors:** Zhuoyu Yang, Peiwen Shi, Kaiting Wang, Min Ma, Yuriko Enomoto, Tomoko Suda, Junyou Li

**Affiliations:** 1 Animal Resource Science Center, Graduate School of Agricultural and Life Sciences, The University of Tokyo, Ago3145 Kasama, Ibaraki 319-0206, Japan; 2 Veterinary Pathophysiology and Animal Health, Graduate School of Agriculture and Life Science, The University of Tokyo, Tokyo 113-8657, Japan

**Keywords:** quebracho tannin, growth performance, meat quality, *A. suum*, growing and finishing swine, free amino acid

## Abstract

Although condensed tannins have shown antiparasitic, antioxidant, and microbiota-modulating effects, their long-term effects on growing–finishing pigs remain unclear. In this study, 24 pigs were randomly assigned at 43 days of age to a control group fed a basal diet or a treatment group supplemented with 0.75% commercial quebracho tannin (MGM-P) for 16 weeks. The effects on *A. suum* infection, growth performance, fecal microbial community structure, and pork quality were evaluated. The results showed that egg counts, adult worm counts, and the number of infected pigs were not significantly different between groups (*p* > 0.1). No differences were observed between groups in growth performance, meat texture, juiciness, or fatty acid composition (*p* > 0.1). Denaturing gradient gel electrophoresis (DGGE) analysis indicated altered fecal microbial community structure in the MGM-P group. The longissimus dorsi from the MGM-P group showed lower lightness and hue angle (*p* < 0.05), and free amino acids associated with bitter taste, including valine and leucine, also tended to decrease (0.05 < *p* < 0.1). These findings indicate that long-term supplementation with 0.75% MGM-P did not influence growth performance or overall meat quality in growing–finishing pigs; its efficacy against *A. suum* infection was limited under the natural infection conditions of this study.

## Simple Summary

With increasing attention to sustainable livestock production, phytogenics are increasingly being explored as feed additives to reduce reliance on synthetic drugs. Condensed tannins are polyphenolic compounds widely distributed in plants. Their ability to bind dietary nutrients may reduce nutrient availability, but they also exhibit antiparasitic, antioxidant, and antimicrobial activities. *A. suum* infection remains a worldwide concern during the growing–finishing period. Continuous tannin supplementation may help avoid withdrawal periods associated with conventional anthelmintics and potentially reduce selection pressure for anthelmintic resistance. In this study, 0.75% quebracho tannin was supplemented in the diets of pigs from 43 days of age for 16 weeks to evaluate its effects on *A. suum* infection, growth performance, pork quality, and fecal microbial communities. The results indicated that quebracho tannins did not alleviate the infection burden of *A. suum*. However, long-term supplementation did not have any negative effects on the animals’ growth performance, the physical properties of the meat, and fatty acid composition. In addition, tannin supplementation altered microbial community structure, reduced meat lightness and showed a tendency toward increased redness; free amino acids associated with bitter taste also tended to decrease.

## 1. Introduction

In recent years, with the growing emphasis on sustainable livestock production, phytogenics, broadly defined as plant-derived bioactive compounds, have attracted increasing attention as feed additives in the swine industry. These compounds possess diverse biological activities and therefore have the potential to contribute to improving animal health while reducing reliance on conventional pharmacological interventions [1,2]. Tannins are naturally occurring polyphenolic secondary metabolites that are widely distributed throughout the plant kingdom and occur abundantly in various plant tissues, including leaves, fruits, and bark [3,4,5]. Based on their molecular structures, tannins are mainly divided into hydrolysable tannins (HT) and condensed tannins (CT) [6]. Traditionally, tannins were regarded as antinutritional factors in monogastric animal nutrition, as the high density of phenolic hydroxyl groups in tannin molecules tends to bind with dietary nutrients—including proteins, polysaccharides and minerals—potentially impairing nutrient digestion and utilization [7,8,9,10]. However, an increasing number of studies have demonstrated that appropriate tannin supplementation can exert beneficial biological effects, including astringent, antidiarrheal, antioxidant, anti-inflammatory, antipathogenic, and gut microbiota-modulating activities [10,11,12,13,14]. Therefore, determining appropriate feeding strategies is important for maximizing the beneficial effects of tannins while minimizing their potential antinutritional effects on animal performance.

At present, research on tannins in the swine industry has primarily focused on weaned piglets, particularly their astringent, antibacterial, antidiarrheal, and antioxidant properties, with potential to reduce antibiotic use in feed [10,12,14]. Studies on the long-term use of tannins in growing–finishing pigs have mainly focused on growth performance, meat quality, and intestinal health [15,16,17]. However, during this stage, infection with *Ascaris suum* (*A. suum*) remains a major parasitic challenge in swine production worldwide [18]. The adverse effects of infection include reduced growth performance and hepatic lesions such as milk spots, which can increase carcass condemnation at slaughter and may affect host susceptibility to other pathogens, imposing a significant economic burden on swine farms [19,20]. Although anthelmintic drugs are effective against *A. suum*, their potentially limited efficacy against migrating larvae and eggs may hinder sustained control of infection. Furthermore, the emergence of anthelmintic resistance and withdrawal periods may further compromise long-term control. Therefore, persistent infection and reinfection remain challenges for effective control of *A. suum* in swine farms.

CTs are polymers of flavonoid units (mainly flavan-3-ols) and are referred to as proanthocyanidins [21]. Due to their plant origin, CTs have relatively low environmental impact and residue concerns in animal-derived products, suggesting their potential for sustainable long-term application in livestock production [22]. They have shown potential as natural anthelmintic agents against gastrointestinal nematodes in ruminants [23,24,25]. In addition, Williams et al. demonstrated the direct anthelmintic activity of CTs against *A. suum* by in vitro tests [26]. However, whether dietary supplementation with CTs can reduce the parasite burden in naturally infected pigs remains unclear. Quebracho tannin, which is one of the CTs abundant in the wood of *Schinopsis lorentzii* native to South America, has been widely studied in animal nutrition worldwide due to their high tannin content and stable supply [16,27]. At present, studies investigating the long-term effects of quebracho tannin supplementation in growing–finishing pigs remain limited. Our laboratory has investigated the commercial quebracho tannin product, MGM-P, in multiple studies, examining its effect on the growth performance and health status of weaned piglets [10,14,28], with the findings indicating that the biological effects of MGM-P may be dose-dependent: 0.5% supplementation showed relatively favorable effects on growth performance, whereas 1% supplementation was associated with adverse effects. In the absence of established in vivo dose information for the anthelmintic efficacy of CTs against *A. suum* in pigs, 0.75% MGM-P was selected as an intermediate inclusion level for long-term supplementation to balance its potential anthelmintic efficacy with the risk of adverse effects on growth performance.

Therefore, the present study investigated the effects of continuously feeding quebracho tannin to growing–finishing pigs starting at 6 weeks of age until body weight close to 110 kg, under natural rearing conditions without the use of additional anthelmintics, on the *A. suum* infection burden, and further examined its effects on growth performance, meat quality characteristics and gut microbiota composition. The objective is to comprehensively evaluate the biological effects of CTs under long-term feeding conditions in growing–finishing pigs and to provide a basis for their potential long-term use as phytogenic feed additives.

## 2. Materials and Methods

### 2.1. Tannins

A commercial MGM-P supplement containing more than 50% quebracho tannin extract was used in this study (Table 1). The product was purchased from Kawamura Co., Ltd. (Tokyo, Japan) and manufactured by UNITAN (Buenos Aires, Argentina).

### 2.2. Animals and Experimental Design

The experimental procedures were carried out at the Animal Resource Science Center of the University of Tokyo (Kasama, Japan) and were approved by the Animal Care and Use Committee of the Faculty of Agriculture, The University of Tokyo (P22-027). All procedures were conducted in accordance with the guidelines for animal experimentation of the University of Tokyo.

In total, 24 piglets (Duroc × Landrace × Yorkshire; 12 males and 12 females) at 43 days of age with an initial average body weight of 26.97 ± 0.59 kg were derived from three sows that were purchased in Nakamura Chikusan (Ibaraki, Japan), and the males were castrated at 1 week of age. All piglets were divided into the treatment group (MGM-P group) and the control group according to their initial weight, sex, and maternal line in a randomized complete group design (Experimental Animal Allocation Procedure v1.1), with 4 piglets per pen and 3 pens in each group. The control group was fed a basal diet. The MGM-P group received a basal diet with 7.5 g/kg MGM-P. To minimize potential environmental effects associated with pen location, the pens were arranged alternately between the control and treatment groups. The experimental period lasted 16 weeks.

### 2.3. Diets and Animal Management

Two dietary phases were devised to cater to the nutritional demands of pigs during different growth stages. The formulation of basal diets adhered to the recommendations outlined by the National Research Council (NRC 2012) to either meet or exceed the nutritional requirements stipulated for pigs. The details of the composition and nutritional levels of growing phase (6 weeks to 14 weeks of age) and finishing phase (14 weeks to 22 weeks of age) diets provided by Marubeni Nisshin Feed (Tokyo, Japan) are presented in Table 2. For both the control group and the MGM-P group, feeds were meticulously prepared through individual mixing using an industrial blender for 30 min. The pigs were housed in six individual semi-enclosed pens with a history of *A.suum* infection, without temperature control. Each pen raised four pigs (a–d), allowing them unrestricted access to feed and water through a 4-hole stainless-steel feeder and a stainless-steel nipple waterer. Daily feces cleaning was implemented. Additionally, on day 32 of the experiment, all pigs received a vaccination against swine fever using Swivac C (Kyoritsu Seiyaku Corporation, Tokyo, Japan). No anthelmintic drugs were administered during the experimental period, and no experimental parasite challenge was performed.

### 2.4. Growth Performance Measurements

During the experimental period, the body weight (BW) and feed intake of animals were recorded at 6 and 22 weeks of age at 10:30 a.m. BW was measured individually for each piglet, whereas feed intake was averaged at the pen level. Average daily gain (ADG), average daily feed intake (ADFI), and the Gain-to-Feed Ratio (G:F) were then calculated. For data analysis, individual pigs were considered the experimental unit for BW and ADG, whereas pens were considered the experimental unit for ADFI and G:F.

### 2.5. Fecal Sampling

Fecal samples were collected rectally using sterile swabs at 6, 10, 14, 18, and 22 weeks of age. The collected fecal samples were divided into two portions: one portion was transferred into 10 mL centrifuge tubes and immediately stored at −20 °C for DNA extraction, while the other portion was kept fresh for fecal egg count analysis.

### 2.6. Fecal Flora Analysis

#### 2.6.1. DNA Extraction

DNA from fecal samples was extracted using the QIAamp Fast DNA Stool Mini Kit (Qiagen, Tokyo, Japan) according to the manufacturer’s instructions. In this experiment, 200 mg of fecal sample was weighed for extraction.

#### 2.6.2. Polymerase Chain Reaction-Denaturing Gradient Gel Electrophoresis (PCR-DGGE) Analysis

PCR-DGGE was used to assess microbial communities in feces. The bacterial 16S rRNA gene V3 region was amplified by PCR using primers 341F-CG (5′-CGCCCGCCGCGCGCGCGGCGGGGCGGGGCGGGGGCACGGGGGGGGCCTACGGGGAGGCAGCAG-3′; GC-clamp is in boldface) as well as 534r (5′-ATTACCGCGGGCTGCTGG-3′) [29]. PCR was performed with a GeneAmp PCR system 9700. The final PCR reaction mixture (25 μL total volume) contained 2.5 μL of 10× PCR Buffer, 1.5 μL of 2.5 mM dNTP, 0.25 μL of each primer (at 100 μM), 0.15 μL of 5 unit/μL TaKaRa Taq polymerase (TaKaRa Bio, Ohtsu, Japan), and 0.5 μL of DNA template. The amplification conditions were 95 °C for 10 min, followed by 30 cycles of 30 s denaturation at 93 °C and 30 s annealing at 65 °C in the first 10 cycles, then at 60 °C in the second 10 cycles and finally at 55 °C in the last 10 cycles. Each annealing step was followed by a 1 min extension at 72 °C, except for the final extension at 72 °C for 5 min. PCR products were confirmed by 1% agar gel electrophoresis.

PCR amplifications were separated using DGGE, which was conducted by the method of Muyzer et al. [30], and used the equipment of the DCode™ Universal Mutation Detection System (Bio-Rad Laboratories, Tokyo, Japan). The conditions of electrophoresis were 60 °C, 100 V for 720 min. Electrophoresis buffer (1× TAE) was maintained throughout at 60 °C. Gels were then stained using SYBR^®^ Gold Nucleic Acid Gel Stain (Thermo Fisher Scientific, Eugene, USA) for 25 min. Gels were visualized and photographed using Ex Flash Light and Ex Flash Light Stand (Bio Tools Inc, Takasaki, Japan).

#### 2.6.3. Analysis of Bacterial DGGE Banding Profiles

DGGE banding patterns were examined with the assistance of GelAnalyzer software (version 19.1) to identify and evaluate individual bands. The number of bands per sample in each DGGE was counted to assess the diversity of gut flora. In addition, similarities in DGGE profile banding patterns were assessed using a presence-and-absence approach, and the results were expressed as a similarity coefficient. The Dice similarity coefficient was employed in this study for pairwise comparisons of DGGE fingerprint profiles. This coefficient is computed using the formula: Dsc = [2j/(a + b)], where a = number of DGGE bands in lane 1, b = number of DGGE bands in lane 2, and j = number of common DGGE bands in lane 1 and lane 2, and Dsc = 1 indicates identical profiles [31]. The calculated similarity coefficients were classified into two categories: within-group similarity and between-group similarity. Within-group similarity was calculated by pairwise comparisons of DGGE profiles among pigs receiving the same feeding treatment, while between-group similarity was calculated by pairwise comparisons of DGGE profiles among pigs receiving different feeding treatments. These two categories were compared to evaluate whether DGGE profiles were more similar within the same dietary treatment than between different dietary treatments, thereby assessing the potential influence of MGM-P supplementation on gut microbial profiles.

### 2.7. A. suum Infection Analysis

#### 2.7.1. Egg Excretion

Fecal egg counting was performed using the McMaster method [32] to analyze the quantity of Ascaris lumbricoides eggs in the feces and intestinal contents. In total, 2.0 g of feces were weighed using a digital scale, and 28 mL of flotation solution (saturated saline, specific gravity 1.2) was added. The samples were mixed well and sieved twice; the filtrate was immediately transferred using an eyedropper or transfer pipette into the two chambers of a McMaster slide. The slide was left to stand for 5 min, and the eggs were then counted using a compound microscope.

#### 2.7.2. Egg Content of Intestinal Contents

After the feeding trial, all the pigs were euthanized. Deep anesthesia was induced with thiopental sodium (Ravonal 0.5 g; Mitsubishi Tanabe Pharma, Osaka, Japan) injected into the jugular vein before dissection. Then, the contents of the duodenum, jejunum, ileum, cecum, colon and rectum were collected in Eppendorf tubes and stored at 4 °C. Egg counting in intestinal contents was performed using the same procedure as for feces.

#### 2.7.3. Adult Worm Burden

After dissecting all the pigs (*n* = 24) and collecting the intestinal contents as described above, the intestine was divided into five locations: duodenum, jejunum, ileum, cecum, colon and rectum. The intestinal cavity was delineated and adult *A. suum* were collected. The number of *A. suum* were measured afterwards.

### 2.8. Meat Quality Analysis

#### 2.8.1. Meat Color Test

The coordinates L*, a*, and b* were measured at three different points in the muscle on a transverse section of the longest back muscle, and an average was calculated for each coordinate per animal. These measurements were performed using a Minolta colorimeter (Chroma Meter CR 410; Konica Minolta, Tokyo, Japan). Equation (1) for calculating the difference (△E*ab) between the two groups of meat samples is shown below:

(1) $$△E*ab\;=\;\sqrt{{\left(L2^{*}\;-\;L1^{*}\right)}^{2}+\;{\left(a2^{*}\;-\;a1^{*}\right)}^{2}+\;{\left(b2^{*}\;-\;b1^{*}\right)}^{2}}$$

ΔE*ab between 0 and 0.1 indicates that there is no difference in the color of the meat in the two groups; between 0.2 and 0.4 indicates that the color can only be identified by professionals; between 0.8 and 1.5 indicates that there is a difference in the color of the meat in the two groups, but it is not significant and is considered as a quality control standard; and greater than 3.0 indicates that there is a significant difference in the color of the meat in the two groups, which might lead to consumer complaints due to the difference in the meat color.

In addition, chroma (C*) and hue angle (H*) were calculated. Chroma (C*) represents the intensity or saturation of meat color, with higher values indicating a more vivid color. Hue angle (H*) represents the dominant color tone, with lower values indicating a color closer to pure red. Equations (2) and (3) for calculating chroma and hue angle are as follows:

(2) $$C^{*}=\;\sqrt{a^{2}\;+\;b^{2}}$$

(3) H* = tan^−1^(b*/a*) × (180/π)

#### 2.8.2. Meat Physical Properties and Composition Analysis

The longissimus dorsi (LD) was collected from the left side of the carcass, extending from between the 6th and 7th ribs to between the 13th and 14th ribs. The samples were stored at −80 °C until analysis and subsequently transported to Bureau Veritas Group (Organization registered with the Ministry of Health, Labour and Welfare and accredited under ISO/IEC 17025, Shimane, Japan) for analysis. The physical characteristics of the longissimus dorsi muscle were evaluated in terms of juiciness and texture. Juiciness-related parameters included moisture content, elongation, pressurized water-holding capacity, pressed juice content, and heating loss. Texture parameters, including tenderness, pliability, toughness, and brittleness, were measured using a Tensipresser texture analyzer after heating the meat samples. The fatty acid composition of the longissimus dorsi muscle was determined by gas chromatography (GC), and the results were expressed as a percentage of total fatty acids. Total lipid content in the muscle was determined using the chloroform–methanol extraction method. The fat melting point was determined using the rising melting point method. The free amino acid profile was determined by high-performance liquid chromatography (HPLC), and the results were expressed as mg/100 g of muscle. Values below the quantification limit (1 mg/100 g) were reported as 0.

### 2.9. Statistical Analysis

Experimental data were analyzed using GraphPad Prism 9 (Version 9.5.1, Build 528). The normality of the FEC data was assessed using the Shapiro–Wilk test. As the FEC data were not normally distributed, differences between the control and MGM-P groups were analyzed using a two-tailed Mann–Whitney U test. The number of FEC-positive pigs was compared between groups using Fisher’s exact test. Differences in egg counts among gastrointestinal regions within each treatment group were analyzed using the Friedman test. Normally distributed data were analyzed using an unpaired two-tailed Student’s *t*-test. DGGE band numbers and similarity indices were analyzed using two-way mixed-effects models with restricted maximum likelihood (REML), followed by Šídák’s multiple comparisons test. Treatment, sampling time, and their interaction were included as fixed effects for band number analysis, whereas similarity category (within-group or between-group), sampling time, and their interaction were included for similarity analysis. Individual pigs and pairwise comparisons were treated as subjects for repeated measurements in band number and similarity analyses, respectively. A *p*-value < 0.05 was considered statistically significant, while 0.05 ≤ *p* < 0.10 was considered to indicate a trend toward a difference.

## 3. Results

### 3.1. Growth Performance

As shown in Table 3, there were no significant differences in BW, ADG, ADFI, or G:F between the tannin and control groups during the growing and finishing stages.

### 3.2. Effects of MGM-P Supplementation on A. suum Infection

The results of fecal egg counting (Table 4) showed that *A. suum* eggs began to appear in fecal samples from both groups at 18 weeks of age. No differences in FEC were observed between the control and tannin groups (18 w: *p* = 0.77; 22 w: *p* = 0.90). In addition, no differences were observed in the number of FEC-positive pigs between the control and MGM-P groups at either 18 or 22 weeks of age (*p* > 0.99).

**Figure 1:**
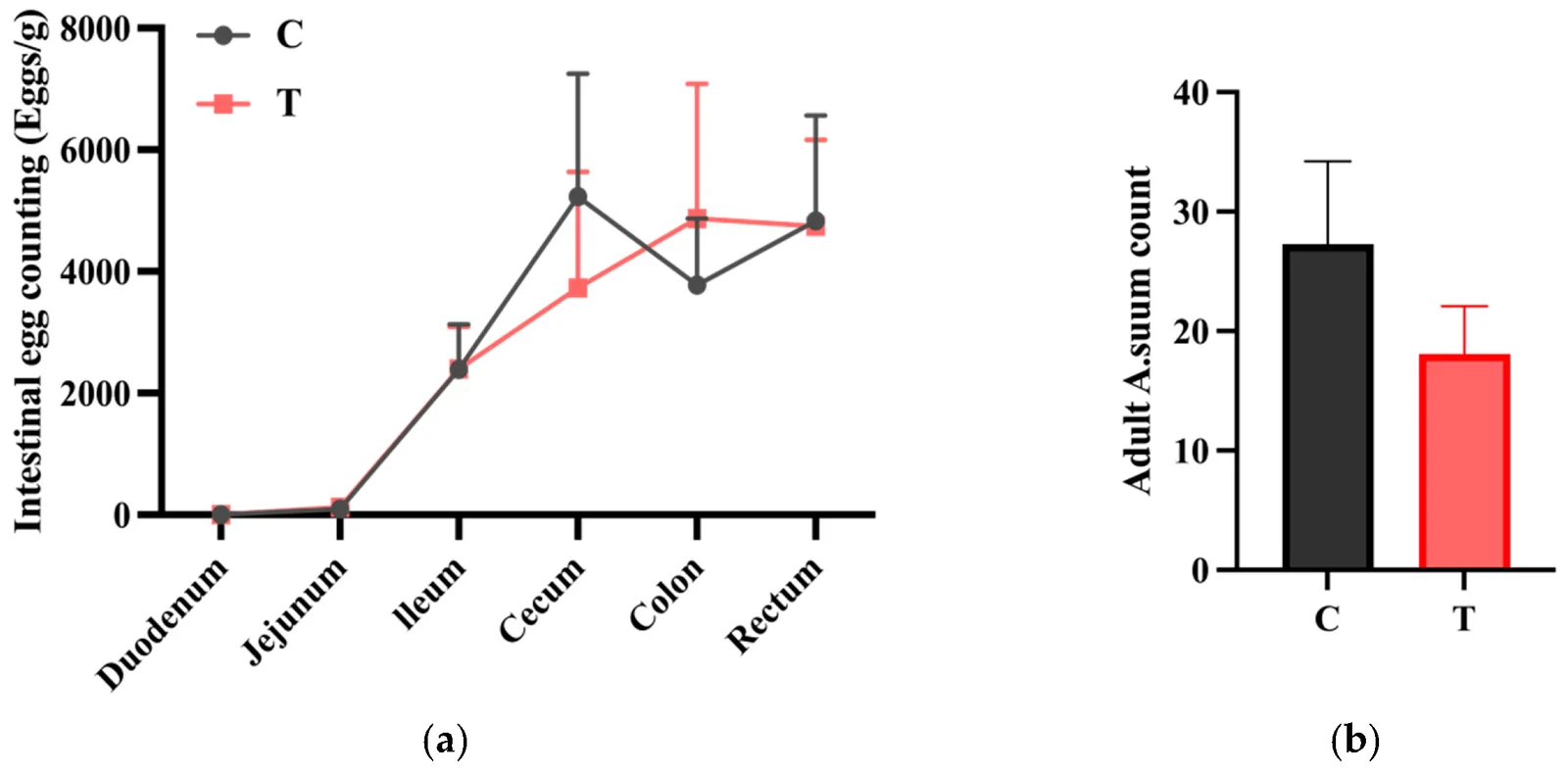
*A. suum* egg (**a**) and adult worm (**b**) burdens in the gastrointestinal tract. C, basal diet; T, basal diet supplemented with 7.5 g/kg MGM-P. Data were analyzed using a two-tailed Mann–Whitney U test and are presented as means ± SEM (*n* = 12). Differences in egg counts among gastrointestinal regions within each treatment group were analyzed using the Friedman test, followed by Dunn’s multiple comparisons test.

Intestinal contents were collected to assess the distribution of *A. suum* eggs across different intestinal segments. The results (Figure 1a) showed that egg counts were generally higher in the large intestine, with significantly higher counts in the colon and rectum than in the duodenum and jejunum in both groups (*p* < 0.05). The highest egg count was observed in the cecum contents in the control group, whereas the MGM-P group showed the highest egg count in the colon contents. No significant differences in egg counts were observed between the control and MGM-P groups in any corresponding intestinal segment (*p* > 0.1).

Adult *A. suum* worms were recovered and counted from the small intestines at necropsy. As shown in Figure 1b, the adult worm burden was numerically higher in the control group than in the MGM-P group (27 ± 6 vs. 18 ± 4 worms/pig, respectively); however, this difference was not statistically significant (*p* = 0.39).

The results of the comprehensive assessment of *A. suum* infection are shown in Table 5. Although some pigs were negative for *A. suum* eggs in fecal and intestinal content samples, adult *A. suum* worms were recovered from the small intestine of these pigs at necropsy. In contrast, one pig (C1-b) was negative for adult worms but positive for *A. suum* eggs in the feces and intestinal contents (150 EPG in feces at week 16 and 100 EPG in rectal contents). Based on the combined results of fecal and intestinal egg detection and adult worm recovery, all pigs in both the control and tannin groups were considered positive for *A. suum* infection, resulting in a 100% infection rate in both groups.

### 3.3. Microbial Structure Analysis

The results for the number of DGGE bands are shown in Table 6. The number of bands in the tannin group (44.43 ± 2.00) was significantly higher than that in the control group (40.83 ± 1.77) at 14 weeks of age (*p* = 0.01). In addition, the number of bands in the MGM-P group at 22 weeks of age (42.13 ± 1.96) tended to be greater than that in the control group (39.57 ± 2.61; *p* = 0.07).

**Figure 2:**
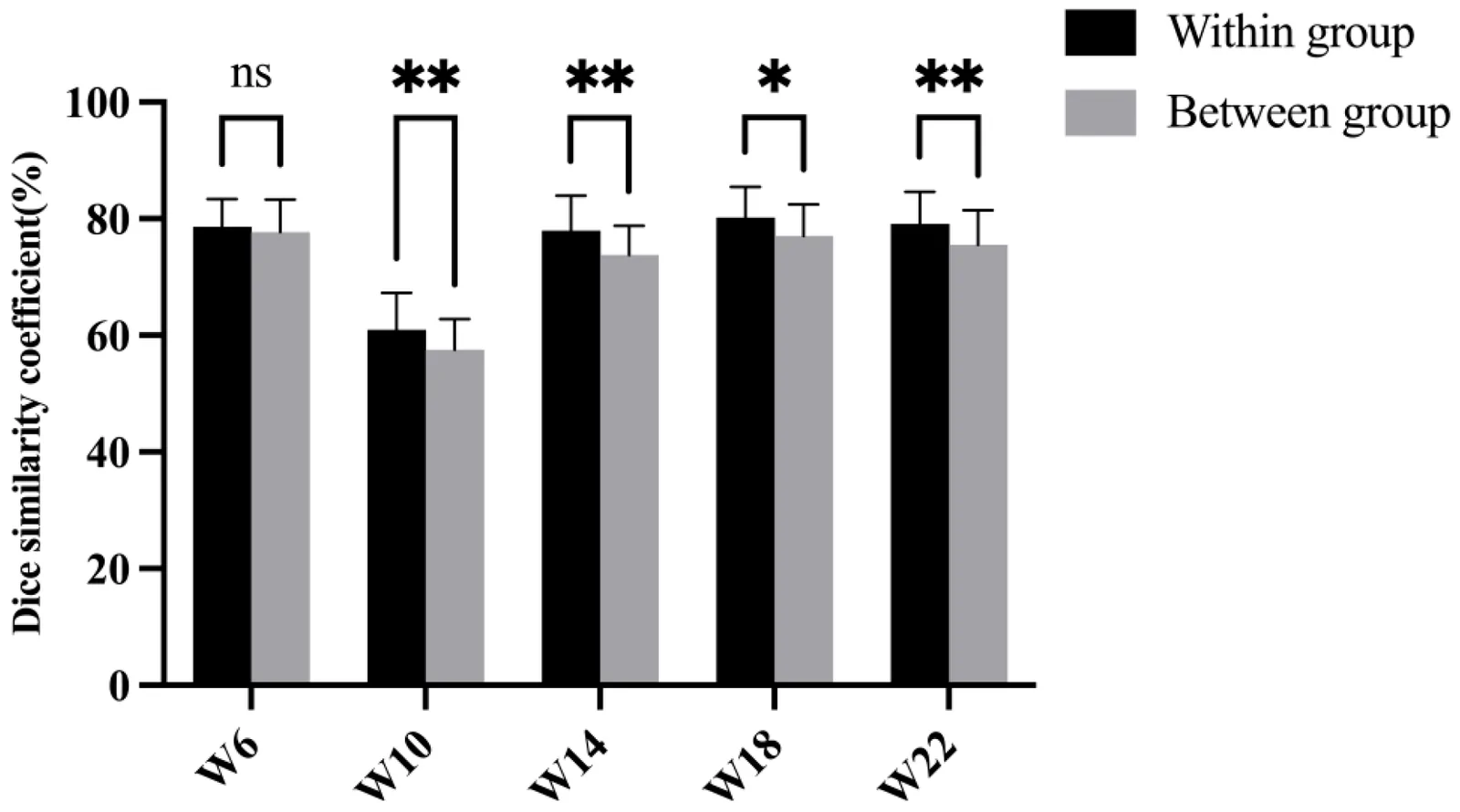
Similarity coefficients of bands between the same and different groups in DGGE gels of grow–finish pigs. W6-22, pigs at 6, 10, 14, 18, and 22 weeks of age. Data were analyzed using a two-way mixed-effects model (REML) followed by Šídák’s multiple comparisons test and are presented as means ± SEM (*n* = 12). *p* < 0.05 was considered statistically significant, and 0.05 ≤ *p* < 0.10 was considered to indicate a trend toward a difference. Asterisks indicate statistical significance: * *p* ≤ 0.05, ** *p* ≤ 0.01.

The band similarity coefficients of DGGE profiles (Figure 2) showed that at the start of the experiment, there were no differences between within-group and between-group similarity. From 10 weeks of age to 22 weeks of age, the band similarity was significantly higher within groups than between groups (*p* < 0.01). In addition, the within-group and between-group similarities decreased at 10 weeks of age compared with 6 weeks of age (60.98 ± 6.29% and 57.58 ± 5.13%, respectively). Then after that, the similar groups increased in the 14, 18, 22 weeks of age by more than 70% (*p* < 0.01).

### 3.4. Meat Quality Attributes

As shown in Table 7, the texture and juiciness in the LD muscle of the MGM-P group were no different from those of the control group. The brittleness of meat in the MGM-P group (1.55) tended to be lower than that in the control group (1.63; *p* = 0.06).

The results of meat color show that the L* value for the meat samples’ color in the MGM-P group was significantly lower than that of the control group (*p* = 0.01). There is no difference in redness (a*) and yellowness (*) of LD between the two groups. The hue angle in the tannin group was significantly lower than that in the control group (*p* = 0.01). The results of the formula for calculating the color differentiation between the two groups of samples showed that the value of ΔE*ab was 1.98, which is between 1.5 and 3.0, indicating that the difference in meat color between the tannin group and the control group is not significant.

### 3.5. Fatty Acid Profiles in the Longissimus Dorsi Muscle

The results in Table 8 show that the composition and amount of saturated fatty acids, monounsaturated fatty acids and polyunsaturated fatty acids in the LD muscle of the MGM-P group were no different from those of the control group (*p* > 0.1).

### 3.6. Free Amino Acid Composition in the Longissimus Dorsi Muscle

Results in Table 9 show that no significant differences were observed in the free amino acid contents between the control and MGM-P groups. Among them, the amounts of lysine, isoleucine, valine and ornithine in the MGM-P group were 11.0 mg/100 g, 4.2 mg/100 g, 5.8 mg/1 mmg, and 0.8 mg/100 g, respectively, which showed a tendency to decrease compared with those in the control group (14.3 mg/100 g, 5.8 mg/100 g, 7.3 mg/100 g, and 1.3 mg/100 g, respectively; 0.05 ≤ *p* < 0.10).

## 4. Discussion

Given that studies on the application of CTs in growing–finishing pigs remain limited, the present study evaluated the effects of a long-term supplementation strategy of MGM-P at an inclusion level of 0.75% on growth performance, *A. suum* infection, intestinal microbiota structure, and meat quality.

The results for growth performance showed no significant differences in ADFI, ADG, and G:F between the two groups, indicating that long-term supplementation with MGM-P for 16 weeks did not affect the growth performance of growing–finishing pigs. Although studies on the effects of tannins on pigs remain limited, it has been widely recognized that tannins can negatively affect ruminant animal appetite and growth. Landau et al. showed that CTs from the quebracho tree reduced feed intake in heifers and suggested that this negative effect was mainly attributable to the astringent taste of CTs and the discomfort associated with short-term ingestion [33]. Similarly, the addition of 3% quebracho tannin significantly reduced feed intake in sheep and poultry, whereas reducing the supplementation level eliminated these negative effects on growth performance [34,35]. Studies on the long-term use of tannins during the growing and finishing stages of pigs have primarily focused on HTs, with a 3% supplementation level showing no significant adverse effects on pig growth performance [15,36]. In the present study, a 0.75% supplementation level of quebracho tannin did not adversely affect growth performance. This suggests that differences in the effects of tannins may be related to the supplementation level and tannin type or source, as well as the animal species and feeding conditions.

Based on the *A. suum* infection status observed during the experiment, under natural infection conditions without the use of any anthelmintics, *A. suum* eggs were found in fecal samples from both groups starting in the 12th week of the experiment. Furthermore, no differences were observed between the two groups in terms of fecal egg shedding or the number of fecal egg-positive pigs. However, the McMaster method has certain limitations in detecting *A. suum* infection, with its sensitivity being lower than that of other diagnostic approaches [37]. To further assess the infection status of the animals, a parasitological examination of the entire gastrointestinal tract was conducted after slaughter. The results showed that *A. suum* eggs were detected in the gastrointestinal contents of nearly all pigs, and adult *A. suum* worms were also recovered. The egg concentration in the gastrointestinal contents was significantly higher than that in fecal samples (3173 ± 762 vs. 160 ± 46 epg, *p* < 0.01). Furthermore, the number of adult worms recovered from the tannin group was lower than that from the control group; however, this difference was not statistically significant, which may be related to considerable inter-individual variation in infection intensity under natural infection conditions. Based on the combined results of fecal egg counts, gastrointestinal egg counts, and adult worm recovery, we concluded that all pigs in this study were infected with *A. suum*. Therefore, the results of this study indicate that long-term feeding of 0.75% MGM-P did not significantly relieve the burden of *A. suum* infection in growing–finishing pigs. Although CTs have been shown in vitro to have a direct anthelmintic effect against *A. suum* larvae, their anthelmintic efficacy varies depending on the chemical structure and plant source [26]. Quebracho tannins, which are characterized by their high tannin content and abundant availability, have been considered promising feed additives for livestock and are commercially available in various products. Although reference to in vivo studies on their efficacy against *A. suum* is still limited, the anthelmintic effects of quebracho tannins have been demonstrated against gastrointestinal nematodes in ruminants, poultry, and mice, and the supplementation levels were over 1% in many in vivo studies [34,38,39]. In addition, Mavri et al. reported that pigs can adapt to dietary tannins by increasing the secretion of proline-rich proteins from the parotid glands, which bind tannins and help protect against their antinutritional effects [40]. This physiological adaptation may further reduce the amount of tannin available to exert direct anthelmintic effects. Therefore, the lack of an anthelmintic effect observed in the present study may be related to the supplementation level and animal species. Further investigations are needed to evaluate the effects of different types of tannins, including MGM-P, at various inclusion levels on *A. suum*.

The DGGE profiles showed that the band similarity between groups was significantly lower than that within groups throughout the experimental period, indicating that supplementation with 0.75% MGM-P altered the fecal microbial community composition of the pigs. The number of DGGE bands also tended to be higher in the MGM-P group than in the control group, suggesting a possible increase in microbial diversity. These findings are consistent with previous studies reporting that tannin supplementation can increase gut microbial diversity in humans, pigs, and other animals [41,42,43,44]. Increased gut microbial diversity is generally considered beneficial for host health and may contribute to maintaining the resilience, resistance, and stability of the microbial ecosystem after environmental stress [45,46]. Ober et al. reported that higher microbial diversity was associated with beneficial effects on disease resistance and growth performance in pigs [47]. However, the increased number of DGGE bands observed in the present study was not accompanied by improved growth performance. Further investigation is needed to identify the bacterial taxa represented by the additional DGGE bands in the MGM-P group to determine whether they have beneficial effects on the host. Since DGGE primarily detects dominant bacterial populations and may fail to detect low-abundance taxa, high-throughput 16S rRNA gene sequencing is needed to comprehensively characterize bacterial community diversity and to identify the bacterial taxa underlying the observed differences.

In terms of physical characteristics, the addition of MGM-P did not significantly alter muscle texture or juiciness. The results on meat color showed that the long-term addition of 0.75% MGM-P significantly reduced the lightness of the meat, and the lower hue angle indicates that the color leaned more toward red. The values of ΔE*ab ranged between 1.5 and 3.0, indicating that the changes in the color of the meat in the two groups were not significant. Kim et al. reported that the amount of myoglobin and myofibrils was higher in the meat of low lightness and high redness than in the meat of high lightness and low redness [48]. Luciano et al. illustrated that quebracho tannins enhanced overall muscle antioxidant capacity, protected myoglobin from oxidation, and increased heme content, thereby maintaining the red color of lamb meat [49,50]. The effect of tannins on pork color may also be related to their antioxidant capacity; further research is needed to investigate the differences in myoglobin and heme levels following tannin supplementation.

In addition, 0.75% MGM-P did not affect the fatty acid composition of the meat. The total lipid content and melting point also showed no significant differences in the LD. Rezae et al. also reported that chestnut tannin supplementation did not influence the fatty acid composition of intramuscular fat, but the proportion of polyunsaturated fatty acids increased in fat tissue [15]. These findings suggest that MGM-P supplementation did not markedly affect fat deposition or the physical and compositional characteristics of LD in the present study. The effects of quebracho tannins on fatty acid composition in adipose tissue warrant further investigation.

In livestock production, free amino acids and peptides in meat are important contributors to its taste characteristics, with their concentrations and compositions contributing to the overall flavor profile and influencing the sensory and nutritional quality of meat [51,52]. According to taste differences, amino acids were divided into umami amino acids, sweet amino acids, bitter amino acids and tasteless amino acids [53]. In this study, the results for free amino acids did not show significant differences between the two groups. Among them, lysine, isoleucine, valine and ornithine showed a downward trend (0.05 < *p* < 0.10). Lysine, isoleucine and valine are considered bitter amino acids, with the last two being recognized as having a strong bitter taste [54]. Their decrease may therefore have a positive effect on the taste of pork. Although ornithine does not elicit a distinct taste at low concentrations, it has been reported to modulate taste responses in rodent models [55]. In addition to their potential effects on taste, lysine, isoleucine, and valine are essential amino acids that must be obtained from the diet. In particular, lysine is the first-limiting amino acid in typical swine diets and plays an important role in muscle protein synthesis [56]. Ornithine is a non-protein amino acid and plays a central role in the urea cycle [57]. Based on the available literature, few studies have evaluated the effects of CT supplementation on free amino acids in pork. Tannins can bind protein and inhibit digestive enzymes and intestinal nutrient transport, thereby potentially reducing protein digestion and amino acid absorption [58,59]. Hidayat et al. reported that feeding high doses of tannins reduced amino acid availability, especially methionine, leucine, and isoleucine in broiler chickens [60]. A study in growing pigs showed that feeding high-tannin sorghum significantly reduced the standardized ileal digestibility of lysine (*p* < 0.001) and decreased plasma urea nitrogen concentrations [61]. In addition, results reported by Cousins et al. also showed that apparent digestibility of proline, glycine and histidine was significantly reduced in pigs fed sorghum diets with high doses of tannin content [62]. Although the differences in free amino acid concentrations between the control and MGM-P groups observed in the present study were not statistically significant, the numerical values for total free amino acids and dipeptides were lower in the MGM-P group; several amino acids showed a downward trend. In addition, our previous study showed a decreased tendency in plasma-free amino acid concentrations in piglets supplemented with 1% MGM-P for 21 days [28]. These results raise concerns about the potential risk of impaired protein utilization during long-term MGM-P supplementation. However, this risk cannot be adequately assessed based on muscle free amino acid alone in the present study. Future studies on long-term tannin supplementation should consider evaluating free amino acid concentrations in both plasma and meat, together with amino acid digestibility, nitrogen balance and serum urea nitrogen to better understand the potential effects of MGM-P on protein utilization.

## 5. Conclusions

Long-term supplementation with 0.75% MGM-P did not adversely affect animal growth performance and meat quality during the growing–finishing period. Moreover, MGM-P supplementation altered meat color and tended to reduce the concentrations of free amino acids associated with bitter taste. DGGE profiles indicated that MGM-P supplementation altered the fecal bacterial community profiles, with a tendency toward an increased number of bands. However, all pigs were infected with A. *suum*, with no significant differences in parasite-related parameters between the two groups, indicating limited efficacy of long-term supplementation with 0.75% MGM-P against *A. suum* under the conditions examined in this study.

## Figures and Tables

**Table 1:** Technical specifications of MGM-P.

Characteristic	Criterion
Polyphenols (mg catechin/g)	>500
Humidity (maximum)	15
pH	4.5 ± 1

**Table 2:** Ingredients and chemical composition of basal diets (as-fed basis).

Items	Growing Phase	Finishing Phase
Ingredients (%) ^(1)^		
Cereals ^(2)^	56	70
Animal products ^(3)^	1	0
Oil seed meal ^(4)^	21	13
Bran ^(5)^	7	14
Others ^(6)^	15	3
Chemical composition ^(7)^		
DM (%)	87.5	87.9
CP (%)	16.4	13.6
EE (%)	5.6	3.3
CF(%)	3.0	3.3
Ash (%)	4.2	4.2
NFE (%)	58.3	63.5
GE (Mcal/kg) ^(8)^	4.10	3.95

^(1)^ Ingredients are provided by Marubeni Nisshin Feed Co., Ltd., Tokyo. ^(2)^ The composition included corn, wheat, wheat flour, bread flour, corn starch, potato starch, dextrin, climacteric corn, milo, rice, low-quality wheat flour, cassava and barley. ^(3)^ The composition included dried whey, mixed meal of pork and chicken ingredients, fish meal, plasma protein and concentrated whey protein. ^(4)^ The composition included soybean oil residue, flaxseed oil residue, rape seed oil residue and corn jam meal. ^(5)^ The composition included corn gluten feed, rice bran, wheat bran and distillers dried grains with soluble. ^(6)^ The composition included confectionery waste, specific animal fats, dextrose, lignocellulosic, calcium phosphate, salt, beet bulb, calcium carbonate, citric acid, dried yeast cell wall, whole egg powder, lactic acid bacteria, baker’s yeast, silicic acid, oligosaccharides, ginkgo biloba extract, garlic extract, Chenopodiaceous extract, licorice extract, pine needle extract, hawthorn extract, butyric acid bacteria, saccharification bacteria, brewer’s yeast, aluminum silicate, galactooligosaccharide, molasses and yucca extract. ^(7)^ DM, dry matter; CP, crude protein; EE, ether extract (crude fat); CF, crude fiber; Ash, crude ash; NFE, nitrogen-free extract; GE, gross energy. ^(8)^ The GE was calculated with the following equation: GE (Mcal⁄kg) = [5.67 × CP(%) + 9.68 × EE(%) + 4.25 × NFE(%) + 4.90 × CF(%)]∕100.

**Table 3:** Effects of dietary MGM-P supplementation on growth performance in grow–finish pigs.

Item	C	T	SEM	*p*-Value
Initial BW (kg)	14.79	14.37	0.39	0.59
Final BW (kg)	106.36	107.09	1.83	0.98
ADG (kg)	0.82	0.83	0.02	0.92
ADFI (kg)	2.24	2.16	0.06	0.61
G:F	0.36	0.38	0.01	0.38

C, control group fed a basal diet; T, treatment group fed a basal diet supplemented with 7.5 g/kg MGM-P. Initial and final body weight (BW) and average daily gain (ADG) were evaluated at the individual pig level (*n* = 12), whereas average daily feed intake (ADFI) and Gain-to-Feed Ratio (G:F) were evaluated at the pen level (*n* = 3). All data were analyzed using an unpaired two-tailed Student’s *t*-test assuming equal variances. *p* < 0.05 was considered statistically significant, and 0.05 ≤ *p* < 0.10 was considered to indicate a trend toward a difference.

**Table 4:** Fecal *A. suum* egg shedding and number of egg-positive pigs during the feeding period.

Week of Age	C		T	
	FEC (epg)	Positive Pigs (n/N)	FEC (epg)	Positive Pigs (n/N)
W6	0	0/12	0	0/12
W10	0	0/12	0	0/12
W14	0	0/12	0	0/12
W18	325 ± 109	8/12	396 ± 138	9/12
W22	133 ± 48	7/12	188 ± 80	8/12

C, basal diet; T, basal diet supplemented with 7.5 g/kg MGM-P; FEC, fecal egg counting; epg, eggs per gram; W6-22, pigs at 6, 10, 14, 18, 22 weeks of age; n, the number of positive pigs; N, the number of pigs in the group. FEC values are presented as means ± SEM (*n* = 12) and were analyzed using a two-tailed Mann–Whitney U test. The number of positive pigs was compared between groups using Fisher’s exact test.

**Table 5:** Individual detection of *A. suum* eggs and adult worms in pigs during the feeding period and at slaughter.

No.	Egg Counting		*A. suum*	Infection ^(1)^
	Feces	Intestine		
C group				
1-a	+	+	+	+
1-b	+	+	-	+
1-c	-	+	+	+
1-d	+	+	+	+
2-a	-	-	+	+
2-b	+	+	+	+
2-c	-	+	+	+
2-d	-	+	+	+
3-a	+	+	+	+
3-b	+	+	+	+
3-c	+	+	+	+
3-d	-	+	+	+
T group				
1-a	+	+	+	+
1-b	+	+	+	+
1-c	-	+	+	+
1-d	+	+	+	+
2-a	+	+	+	+
2-b	-	+	+	+
2-c	+	+	+	+
2-d	+	+	+	+
3-a	+	+	+	+
3-b	-	+	+	+
3-c	-	+	+	+
3-d	+	+	+	+

C, basal diet; T, basal diet supplemented with 7.5 g/kg MGM-P; 1–3, 3 pens in each group; a–d, 4 pigs in each pen. +, positive result; -, negative result. ^(1)^ Final infection status was comprehensively determined based on the combined results of fecal and intestinal *A. suum* egg detection and adult worm recovery.

**Table 6:** Band numbers of control and MGM-P groups in DGGE gels of grow–finish pigs.

Weeks of Age	C	T	SEM	*p*-Value
W6	40.5	39.43	1.13	0.38
W10	51.71	53.22	0.81	0.37
W14	40.83	44.43	0.72	0.01
W18	42.00	44.00	0.74	0.15
W22	39.57	42.13	0.70	0.07

C, basal diet; T, basal diet supplemented with 7.5 g/kg MGM-P; W6-22, pigs at 6, 10, 14, 18, 22 weeks of age. Data were analyzed using a two-way mixed-effects model (REML) followed by Šídák’s multiple comparisons test.

**Table 7:** Effects of dietary MGM-P supplementation on physical characteristics and meat color in LD muscle of grow–finish pigs.

	Treatments			
Items	C	T	SEM	*p*-Value
Texture				
Tenderness, ×10^4^ gf/cm^2^	3.63	3.90	0.019	0.52
Pliability	1.45	1.49	0.02	0.44
Toughness, ×10^7^ gf/cm^2^*cm	6.45	8.96	0.19	0.34
Brittleness	1.63	1.55	0.02	0.06
Juiciness				
Moisture Content, %	72.1	72.3	0.21	0.70
Elongation, cm^2^/g	13.1	13.6	0.39	0.59
Pressurized Water-holding Capacity, %	76.7	76.1	0.68	0.70
Pressed Juice Content, %	42.5	42.1	0.59	0.73
Heating loss, %	23.9	24.1	0.89	0.94
Meat color				
L*	40.9	39.24	0.33	0.01
a*	4.41	5.46	0.34	0.12
b*	2.61	2.35	0.12	0.29
H* ^(1)^	33.63	24.57	1.90	0.01
C* ^(2)^	5.23	6.00	0.32	0.23
ΔE*ab ^(3)^	1.98			

C, basal diet; T, basal diet supplemented with 7.5 g/kg MGM-P; L*, lightness; a*, redness; b*, yellowness; H*, hue angle; C*, chroma. ^(1)^ The formula for calculating hue angle: H* = tan^−1^(b*/a*) × (180/π). ^(2)^ The formula for calculating chroma: $C^{*}=\;\sqrt{a^{2}\;+\;b^{2}}$. ^(3)^ The formula for calculating the difference: $△E*ab\;=\;\sqrt{{\left(L2^{*}\;-\;L1^{*}\right)}^{2}+\;{\left(a2^{*}\;-\;a1^{*}\right)}^{2}+\;{\left(b2^{*}\;-\;b1^{*}\right)}^{2}}$. Data were analyzed using an unpaired two-tailed Student’s *t*-test assuming equal variances. *p* < 0.05 was considered statistically significant, and 0.05 ≤ *p* < 0.10 was considered to indicate a trend toward a difference.

**Table 8:** Effects of dietary MGM-P supplementation on fatty acids in LD of grow–finish pigs.

	Treatment			
Item	C	T	SEM	*p*-Value
C4:0, %	0.0	0.0	0.0	-
C6:0, %	0.0	0.0	0.0	-
C8:0, %	0.0	0.0	0.0	-
C10:0, %	0.1	0.1	0.0	0.55
C12:0, %	0.1	0.1	0.0	1.00
C14:0, %	1.3	1.4	0.0	0.18
C15:0, %	0.0	0.0	0.0	0.34
C16:0, %	26.7	26.3	0.3	0.58
C17:0, %	0.1	0.1	0.0	1.00
C18:0, %	13.5	13.1	0.3	0.61
C20:0, %	0.2	0.3	0.0	0.29
C22:0, %	0.0	0.0	0.0	-
C24:0, %	0.0	0.0	0.0	-
C14:1, %	0.0	0.0	0.0	-
C16:1, %	3.2	3.4	0.1	0.34
C17:1, %	0.1	0.2	0.0	0.26
C18:1, %	43.9	42.7	0.6	0.32
C18:1 cis-, %	3.8	3.9	0.1	0.33
C18:1 trans-, %	0.0	0.0	0.0	-
C20:1, %	0.8	0.8	0.0	0.69
C22:1, %	0.0	0.0	0.0	-
C24:1, %	0.0	0.0	0.0	-
C18:2(n-6), %	4.2	4.2	0.2	0.94
C18:3(n-6), %	0.1	0.1	0.0	1.00
C20:2(n-6), %	0.1	0.1	0.0	1.00
C20:3(n-6), %	0.0	0.0	0.0	-
C20:4(n-6), %	0.0	0.0	0.0	-
C22:4(n-6), %	0.0	0.0	0.0	-
C18:3(n-3), %	0.1	0.1	0.0	1.00
C20:4(n-3), %	0.0	0.0	0.0	-
C20:5(n-3) EPA, %	0.0	0.1	0.0	0.17
C22:5(n-3), %	0.0	0.0	0.0	0.34
C22:5(n-3) DHA, %	0.0	0.0	0.0	0.34
SFA, %	42.1	41.4	0.7	0.66
MUFA, %	51.8	50.9	0.6	0.47
PUFA, %	4.6	4.7	0.2	0.92
PUFA:SFA ratio	0.7	0.7	0.0	0.99
UFA, %	56.4	55.5	0.7	0.55
ω6 FA, %	4.5	4.5	0.2	0.95
ω3 FA, %	0.1	0.2	0.0	0.16
ω6:ω3 PUFA ratio	0.1	0.1	0.0	0.82
Total lipids, g/100 g	2.8	2.7	0.3	0.81
Fat melting point, ℃	33.5	35.0	0.5	0.19

C, basal diet; T, basal diet supplemented with 7.5 g/kg MGM-P; SFA = saturated fatty acid; MUFA = monounsaturated fatty acid; PUFA = polyunsaturated fatty acid. Data were analyzed using an unpaired two-tailed Student’s *t*-test assuming equal variances. *p* < 0.05 was considered statistically significant, and 0.05 ≤ *p* < 0.10 was considered to indicate a trend toward a difference.

**Table 9:** Effects of dietary MGM-P supplementation on amino acids in LD of grow–finish pigs.

	Treatment			
Item, mg/100 g	C	T	SEM	*p*-Value
Umami and Sour Tasting Amino Acids				
Aspartic acid	2.8	2.5	0.1	0.26
Glutamic acid	18.3	19.0	1.2	0.80
Glutamine	33.8	29.5	3.0	0.51
Asparagine	2.7	2.0	0.2	0.21
Total	57.3	52.5	3.9	0.58
Sweet and Slightly Sweet Tasting Amino Acids				
Glycine	14.8	12.7	0.7	0.16
Alanine	24.8	22.8	1.4	0.51
Threonine	5.2	4.3	0.4	0.28
Serine	6.8	4.7	0.6	0.10
Proline	6.0	5.2	0.5	0.41
Total	57.7	53.0	3.9	0.60
Flavor and Bitter-Tasting Amino Acids				
Methionine	6.2	4.3	0.6	0.14
Lysine	14.3	11.0	0.9	0.06
Isoleucine	5.8	4.2	0.5	0.09
Leucine	13.3	9.8	1.1	0.12
Phenylalanine	9.0	6.3	0.8	0.12
Tyrosine	3.8	2.7	0.2	0.17
Valine	7.3	5.8	0.4	0.07
Histidine	3.0	2.5	0.2	0.17
Arginine	24.7	19.3	1.9	0.19
Cysteine	0.0	0.0	0.0	-
Total	79.5	66.0	7.5	0.412
Other amino acids (specific functionality)				
Taurine	28.7	25.7	2.9	0.65
Ornithine	1.3	0.8	0.1	0.09
GABA (γ- Aminobutyric Acid)	0.0	0.0	0.0	-
Total	29.8	26.5	3.1	0.61
Dipeptide				
Carnosine	656.3	611.2	38.4	0.60
Anserine	25.8	25.2	1.7	0.86
Total free amino acids	232.5	194.5	14.1	0.21
Total amino acids constituting peptide	940.0	861.3	36.1	0.32

C, basal diet; T, basal diet supplemented with 7.5 g/kg MGM-P. Data were analyzed using an unpaired two-tailed Student’s *t*-test assuming equal variances. *p* < 0.05 was considered statistically significant, and 0.05 ≤ *p* < 0.10 was considered to indicate a trend toward a difference.

## Data Availability

The data supporting the findings of this study are available from the corresponding author upon reasonable request.

## Abbreviations

The following abbreviations are used in this manuscript:

ADFI	Average daily feed intake
ADG	Average daily gain
Ash	Crude ash
*A. suum*	*Ascaris suum*
a*	Redness
BW	Body weight
b*	Yellowness
C	Control group
C*	Chroma
CF	Crude fiber
CTs	Condensed tannins
CP	Crude protein
DGGE	Denaturing gradient gel electrophoresis
DM	Dry matter
G:F	Gain-to-Feed Ratio
GE	Gross energy
H*	Hue angle
HTs	Hydrolysable tannins
LD	Longissimus dorsi
L*	Lightness
MUFA	Monounsaturated fatty acid
NFE	Nitrogen-free extract
PUFA	Polyunsaturated fatty acid
SEM	Standard Error of the Mean
SFA	Saturated fatty acid
T	Treatment group
